# Association between 24-h movement behaviors and psychological distress in older adults with chronic diseases: a compositional isotemporal substitution analysis

**DOI:** 10.3389/fpsyg.2026.1782087

**Published:** 2026-05-19

**Authors:** Hanchen Tian, Hongyu Si, Hong Wang, Lin Wang

**Affiliations:** 1 School of Kinesiology and Physical Education, Zhengzhou University, Zhengzhou, China; 2 School of Wushu, Wuhan Sports University, Wuhan, China; 3 School of Physical Education, Wuhan University of Technology, Wuhan, China

**Keywords:** 24-h movement behaviors, chronic disease, isotemporal substitution, older adults, psychological distress

## Abstract

**Objective:**

This study aimed to investigate the association between 24-h movement behavior patterns and psychological distress in older adults with chronic diseases using compositional isotemporal substitution analysis.

**Methods:**

This cross-sectional study included a final analytic sample of 143 community-dwelling older adults aged 60 years and above with chronic diseases. Twenty-four-hour movement behaviors and psychological distress were measured using an ActiGraph wGT3X-BT accelerometer and the Kessler Psychological Distress Scale (K10), respectively. Isotemporal substitution analyses were used to estimate associations between time reallocations among 24-h movement behaviors and psychological distress.

**Results:**

Participants had a mean age of 69.94 ± 4.95 years and a mean K10 score of 19.55 ± 6.16. After adjusting for confounding factors, the proportion of moderate-to-vigorous physical activity (MVPA) within the 24-h composition was significantly negatively associated with K10 (*B* = −2.96, *p* = 0.011), and light-intensity physical activity (LPA) was also negatively associated with K10 (*B* = −2.53, *p* < 0.001). In contrast, sedentary behavior (SB) was significantly positively associated with K10 (*B* = 5.11, *p* < 0.001), whereas sleep was not significant (*p* = 0.831). Isotemporal substitution analysis further indicated that reallocations toward MVPA showed the strongest favorable associations. Replacing 15 min of SB or sleep with MVPA was associated with lower K10 scores, with 95% CIs excluding zero, particularly for SB-to-MVPA substitution (*B* = −0.92, 95% CI: −1.41 to −0.43). Reallocating time away from MVPA was associated with higher K10 scores, whereas SB-to-LPA substitution showed smaller but significant favorable associations. Substitutions between sleep and SB were not significant.

**Conclusion:**

The 24-h time distribution of movement behaviors in older adults with chronic diseases was closely associated with psychological distress. Higher relative MVPA and lower relative SB were associated with more favorable K10 profiles, while reallocating SB to LPA showed a smaller but still meaningful association.

## 1 Introduction

Global population aging has become one of the most prominent demographic transitions of the 21st century, accompanied by a sharp increase in the prevalence of chronic non-communicable diseases (e.g., cardiovascular diseases, diabetes, and arthritis) (Balashova et al., 2022). Chronic diseases not only impair physiological function in older adults but also pose long-term and severe challenges to mental health. Unlike generally healthy older adults, those with chronic diseases must cope not only with the physical pain and functional decline caused by their illness disease but also with the resulting psychological stress (Palmese et al., 2025). Specifically, physiological mechanisms, wherein chronic pain, inflammation, and metabolic disturbances (e.g., insulin resistance) disrupt neuroendocrine homeostasis, can lead to the dysregulation of critical neurotransmitters such as serotonin and dopamine (Berk et al., 2013). Additionally, functional decline, characterized by reduced mobility and independence, erodes self-esteem and sense of control, often necessitating reliance on external care. Furthermore, socio-cognitive factors compound these issues; activity limitations foster social isolation and loneliness, while negative cognitive appraisals, including fear of disability and perceived burdensomeness, trigger and sustain psychological distress (Holm et al., 2023). Psychological distress refers to a range of non-specific negative emotional experiences, such as depressed mood, anxiety, tension, and emotional dysregulation. A substantial body of research has demonstrated that older adults with chronic diseases are at high risk for psychological distress, including depression and anxiety (Lin et al., 2021; Werneck and Stubbs, 2024). Persistent pain, functional limitations, social isolation, and uncertainty about the future jointly influence psychological status, substantially reducing quality of life and increasing caregiving burdens on families and society (Santini et al., 2020). Therefore, identifying modifiable behavioral correlates associated with lower psychological distress in this population has become an important priority in public health and clinical gerontology.

Older adults commonly exhibit unfavorable lifestyle behaviors such as reduced physical activity, prolonged sedentary time, and insufficient or excessive sleep (Cabanas-Sánchez et al., 2019). Previous meta-analyses have indicated that both structured moderate-to-vigorous exercise and daily light physical activity can effectively reduce depressive symptoms in older adults (Bigarella et al., 2022). Moreover, sedentary behavior (SB) has been identified as an independent risk factor for mental health Studies have found that even when individuals meet physical activity guidelines, excessive sedentary time (e.g., prolonged television viewing) remains significantly associated with higher risks of depression and anxiety (Zhou et al., 2023). In addition, the central role of sleep in maintaining emotional stability has received increasing attention. Among older adults with chronic diseases, sleep disturbances (e.g., insomnia and sleep fragmentation) are highly prevalent and can form a complex bidirectional vicious cycle with daytime negative affect and cognitive decline (Bolstad et al., 2025; Overton et al., 2024). Although these studies provide valuable insights into the relationship between behaviors and mental health, most adopt an “isolated perspective,” examining the association between a single activity type (e.g., exercise or social participation) and psychological distress. However, this approach overlooks a fundamental fact: time in a day is finite and fixed at 24 h. Daily activities constitute a closed “time-use composition”; increasing time spent in one activity necessarily reduces time spent in one or more other activities (Clark and Martins, 2025; Zhang et al., 2023). Therefore, assessing the influence of a single activity in isolation may introduce bias by ignoring what activity is being displaced, thereby limiting the ecological validity and practical value of the study conclusions.

To overcome this limitation, an increasing number of studies in recent years have adopted a holistic perspective to examine 24-h activity patterns (Clark and Martins, 2025; Lai et al., 2024; Wang et al., 2025). The 24-h movement behavior framework integrates physical activity, sedentary behavior, and sleep across the whole day to form a complementary health behavioral portfolio, and this behavioral pattern is of great significance to mental health (Liang et al., 2024b). These behaviors follow the “constant-sum constraint” of compositional data: increasing one behavior necessarily decreases others to keep the total time fixed at 24 h (Dumuid et al., 2019). Although isotemporal substitution models have been successfully applied to analyze associations between 24-h activity patterns and physiological adverse health indicators such as obesity and cardiometabolic outcomes (Cao et al., 2022; Ji et al., 2024), their use for mental health in older adults is emerging. For example, a recent study of urban Chinese older adults used this approach to link self-reported movement behaviors to depressive symptoms (Ji et al., 2025). However, two critical gaps remain. First, much of the existing literature, including that work, has relied on self-report questionnaires (e.g., IPAQ), which are prone to recall bias and systematically misestimate behavior, particularly under-reporting sedentary time (Prince et al., 2020). This highlights a pressing need for studies using objective, device-based measures to provide more accurate quantification. Second, while these studies provide insights into the general older population, the unique challenges faced by older adults with chronic diseases have been largely overlooked. This subgroup’s activity patterns are often constrained by symptoms like pain and fatigue (Wei et al., 2023), making time-reallocation strategies both more difficult to implement and potentially more critical for their wellbeing. It thus remains unclear which objectively measured activity substitutions are associated with more favorable psychological distress profiles in this specific, vulnerable population.

Therefore, using accelerometer-derived data, we applied a compositional and isotemporal substitution framework to examine the association between 24-h movement behaviors and psychological distress in older adults with chronic diseases. This study makes three primary contributions: first, it employs a methodologically robust, device-based approach that overcomes the limitations of self-report; second, it provides targeted evidence for a clinically significant and vulnerable population whose activity is constrained by disease; and third, it extends the outcome of interest from depressive symptoms to the broader construct of psychological distress. Our findings seek to inform the development of more precise, evidence-based behavioral recommendations for this high-risk group.

## 2 Materials and methods

### 2.1 Study design and participants

This study employed a cross-sectional design. Data were collected from September 2024 to June 2025. Convenience sampling was used to recruit community-dwelling older adults from four communities in Wuhan, Hubei Province, China (East Community and West Community of the Mafangshan Campus of Wuhan University of Technology, Youli Community, and Jiangdayuan Community) through health education lectures, recruitment posters, and other approaches. Inclusion criteria were: (1) age ≥ 60 years; (2) residence in the current location for at least 6 months; (3) ability to engage in normal physical activity and communicate verbally; (4) voluntary participation with written informed consent and agreement to wear an accelerometer continuously for 7 days. Exclusion criteria included: (1) major diseases or severe cardiovascular disease leading to inability to perform physical activity, or severe cognitive impairment (Mini-Mental State Examination, MMSE < 18); (2) evident motor impairment or severe visual/hearing impairment; (3) long-term or recent use of psychotropic medications; (4) missing key study data, such as invalid accelerometer data or incomplete questionnaires.

The present study was a restricted analysis based on a broader community-derived dataset and specifically focused on older adults with chronic diseases. Chronic disease status was determined according to participants’ self-reported history of whether they had ever been diagnosed by a physician with any of the listed chronic conditions. Participants reporting at least one of these conditions were included in the final analytic sample. The chronic conditions considered included hypertension, dyslipidemia, diabetes or high blood sugar, cancer or malignant tumor, chronic lung disease, liver disease, heart disease, stroke or cerebrovascular disease, kidney disease, stomach or other digestive disease, and arthritis, rheumatism, or chronic musculoskeletal disease.

Before questionnaire administration, all participants were informed of the study objectives and provided written informed consent. Subsequently, trained researchers guided participants to complete paper-based questionnaires (including demographic information) and measured anthropometric indicators such as height and weight. During questionnaire administration, if a participant had difficulty reading or understanding an item, the researcher provided standardized oral explanations or repeated the item wording when necessary, without giving leading prompts. Participants who were ultimately unable to complete the questionnaire reliably were treated as having incomplete questionnaire data and were excluded from the final analysis. Finally, accelerometers were distributed to participants in batches with detailed instructions on wear protocols and precautions. During the monitoring period, researchers sent reminders every two days to improve wear compliance.

A total of 237 older adults were initially recruited. Of these, 12 participants were excluded for failing to complete the questionnaire and physical examination procedures, and another 15 were further for not meeting the prespecified valid wear-day criteria for the accelerometer. Thus, 210 participants had complete valid data, among whom 143 older adults diagnosed with chronic diseases were included in the final analysis. This study adhered to the principles of the Declaration of Helsinki and was approved by the Ethics Committee of Wuhan Sports University (Approval No.: 2025119). All participants took part voluntarily and could withdraw unconditionally at any stage.

### 2.2 Measurements

#### 2.2.1 24 -h movement behaviors

Twenty-four-hour movement behaviors were objectively measured using a triaxial ActiGraph wGT3X-BT accelerometer (ActiGraph Corp, Pensacola, FL, USA). The epoch length was set to 60 s. Monitoring lasted for a complete and continuous 7 days (including 5 weekdays and 2 weekend days). A valid day was defined as ≥ 10 h of wear time; participants with ≥ 3 valid days (2 weekdays and 1 weekend day) were included (Ars et al., 2026). During the test, participants wore the accelerometer on the non-dominant wrist (except during water-based activities). After completion, ActiLife 6.13.3 software was used to initialize, download, and analyze data. Participants who did not meet the minimum valid wear-time requirement were considered to have invalid accelerometer data and were excluded from the analytic sample. Missing or invalid accelerometer-derived movement behavior data were not imputed, to avoid introducing additional bias into the subsequent compositional analyses. Meanwhile, based on the intensity cut points developed by Freedson, activity intensity was classified as SB (0–99 counts/min), light-intensity physical activity (LPA) (100–1,951 counts/min), and moderate-to-vigorous physical activity (MVPA) (≥ 1,952 counts/min) (Freedson et al., 1998). To improve the accuracy of accelerometer-derived sleep estimates, a paper-based sleep/activity log was also provided to record participants’ daily bedtime and wake-up time. Accordingly, the sleep variable used in this study should be interpreted as an actigraphy-estimated sleep/rest interval based primarily on wrist inactivity, rather than a direct measure of sleep architecture or physiological sleep stages.

#### 2.2.2 Psychological distress

Psychological distress over the past four weeks was assessed using the Kessler Psychological Distress Scale (K10), which measures the frequency of non-specific mental health symptoms such as anxiety and stress. The scale contains 10 items rated on a 5-point Likert scale (1 = “never” to 5 = “always”). Total scores range from 10 to 50, with higher scores indicating more severe psychological distress. Further, scores were categorized into four levels for between-group comparisons: 10–15 indicated low psychological distress (Q1), 16–21 indicated moderate psychological distress (Q2), 22–29 indicated high psychological distress (Q3), and 30–50 indicated very high psychological distress (Q4). These four levels were interpreted as reflecting good, fair, poor, and very poor mental health status, respectively (Andrews and Slade, 2001). The scale has been widely used in China to assess mental health status among rural and community-dwelling older adults (Zhou et al., 2024). In this study, Cronbach’s alpha for the K10 was 0.90, indicating good internal consistency.

#### 2.2.3 Covariates

Based on published research (Duck et al., 2019; Lai et al., 2024; Yeh et al., 2024), selected questionnaire information was used as control variables, including sex (male, female), age, economic status (basically sufficient, relatively affluent), educational level (primary school or below, secondary school, bachelor’s degree or above), social support, and body mass index (BMI). BMI was calculated as BMI = weight (kg)/[height (m)]^2^. The number of chronic conditions was categorized into two groups: one chronic disease, and two or more chronic diseases. Social support was assessed using the Social Support Rating Scale (SSRS) developed by Xiao Shuiyuan, including three dimensions—objective support (3 items), subjective support (4 items), and utilization of support (3 items)—for a total of 10 items. Total scores range from 12 to 64, with higher scores indicating higher levels of social support (Shuiyuan, 1994).

### 2.3 Statistical analysis

Continuous variables were described as mean ± standard deviation, and categorical variables as frequency (percentage). Compositional geometric means and a variation matrix were calculated to summarize the central tendency and proportional dependence of the 24-h movement behaviors (Chastin et al., 2015). Because the data were compositional and constrained to 1,440 min/day, sleep, SB, LPA, and MVPA were expressed as isometric log-ratio (ILR) coordinates before regression modeling (Dumuid et al., 2018). For substitution analyses, the reference composition and each reallocated composition were transformed using the same orthonormal ILR basis and entered into the fitted linear model to obtain predicted K10 values. To enable behavior-specific interpretation, additional pivot-coordinate parameterizations were fitted, in which the first ILR coordinate represented one focal behavior relative to the geometric mean of the remaining three behaviors; the corresponding SBPs, sign matrices (Supplementary Appendix 1; Smithson and Broomell, 2024). Multiple linear regression models were fitted with ILR coordinates as predictors and K10 score as the outcome, adjusting for age, sex, BMI, education, economic status, number of chronic diseases and social support. Regression results are reported as unstandardized coefficients (*B*) and standard errors (SE) (Chen et al., 2025), where *B* represents the expected change in K10 associated with a one-unit increase in the corresponding ILR coordinate, conditional on covariates. *P*-values for individual movement behaviors were obtained from the coefficient test of the first pivot-coordinate ILR term. For isotemporal substitution, effects were defined as the difference between the predicted K10 value for the reallocated composition and that for the reference composition (Dumuid et al., 2019). We first examined 15-min pairwise reallocations, and then assessed dose-response patterns in 5-min increments from −60 to +60 min, subject to the observed compositional support of the least prevalent behavior. Statistical inference for substitution effects was based on 95% confidence intervals; effects whose intervals excluded zero were considered significant. Given the potential influence of social support on both individuals’ activity participation levels and psychological health status (Lindsay Smith et al., 2017; Tengku Mohd et al., 2019), we conducted a sensitivity analysis excluding social support as a covariate to examine whether the main results changed. Analyses were performed in R (version 4.4.2) using the compositions and robCompositions packages, with two-tailed *p* < 0.05 considered statistically significant for model coefficients.

## 3 Results

### 3.1 Participant characteristics

Table 1 presents the characteristics of the 143 older adults with chronic diseases included in this study. The mean age was 69.94 ± 4.95 years, and mean BMI was 23.90 ± 2.93. The mean SSRS score was 37.74 ± 9.82, and the mean K10 score was 19.55 ± 6.16. Group comparisons showed no statistically significant differences in age (*p* = 0.290), BMI (*p* = 0.218), sleep duration (*p* = 0.508), sex distribution (*p* = 0.604), educational attainment (*p* = 0.060), or socioeconomic status (*p* = 0.096). SSRS also differed significantly across the three groups (*p* = 0.036): older adults with lower psychological distress had the highest social support level (40.90 ± 7.22), whereas those with higher psychological distress had the lowest (34.87 ± 8.08). In addition, 24-h movement behaviors showed significant heterogeneity across psychological distress groups. As psychological distress increased (from Group 1 to Group 3), mean LPA and MVPA time decreased significantly (*p* < 0.001), whereas SB time showed a significant increasing trend (*p* = 0.004). Differences in sleep across groups were not significant (*p* = 0.508). No participant in the final analytic sample was classified into the Q4 category (K10 30–50); accordingly, between-group comparisons in Table 1 are presented for Q1–Q3 only.

**TABLE 1:** Basic characteristics of participants.

Variables	Total (*n* = 143)	Q1 (*n* = 31)	Q2 (*n* = 73)	Q3 (*n* = 39)	*p*
Age, mean ± SD	69.94 ± 4.95	68.91 ± 4.13	69.78 ± 5.85	70.98 ± 5.46	0.290
BMI, mean ± SD	23.90 ± 2.93	23.14 ± 2.08	23.98 ± 3.21	24.35 ± 2.91	0.218
LPA, mean ± SD	169.87 ± 71.55	203.86 ± 72.92	177.07 ± 67.67	129.39 ± 59.45	< 0.001
MVPA, mean ± SD	39.92 ± 23.76	55.21 ± 22.93	40.50 ± 23.15	26.70 ± 17.58	< 0.001
Sleep, mean ± SD	495.48 ± 97.03	510.22 ± 100.71	495.90 ± 93.92	482.96 ± 100.62	0.508
SB, mean ± SD	734.73 ± 141.74	670.71 ± 157.62	726.54 ± 125.87	800.94 ± 132.16	< 0.001
SSRS, mean ± SD	37.74 ± 9.82	40.90 ± 7.22	37.93 ± 11.20	34.87 ± 8.08	0.036
K10, mean ± SD	19.55 ± 6.16	11.00 ± 1.46	19.29 ± 2.96	26.85 ± 3.04	< 0.001
Sex, *n* (%)					0.604
Female	62 (43.36)	11 (35.48)	33 (45.21)	18 (46.15)	
Male	81 (56.64)	20 (64.52)	40 (54.79)	21 (53.85)	
Education level, *n* (%)					0.060
Primary school or below	25 (17.48)	3 (9.68)	10 (13.70)	12 (30.77)	
Secondary school	64 (44.76)	13 (41.94)	33 (45.21)	18 (46.15)	
College or above	54 (37.76)	15 (48.39)	30 (41.10)	9 (23.08)	
Socioeconomic status, *n* (%)					0.096
Basically sufficient	71 (49.65)	11 (35.48)	36 (49.32)	24 (61.54)	
Relatively affluent	72 (50.35)	20 (64.52)	37 (50.68)	15 (38.46)	

F, ANOVA; χ^2^, Chi-square test; SD, standard deviation. K10 score categories are defined as follows: Q1 = 10–15, low psychological distress/good mental health; Q2 = 16–21, moderate psychological distress/fair mental health; Q3 = 22–29, high psychological distress/poor mental health; and Q4 = 30–50, very high psychological distress/very poor mental health. No participant in the final analytic sample was classified into Q4; therefore, Table 1 presents comparisons across Q1–Q3 only.

### 3.2 Compositional characteristics of 24-h movement behaviors in older adults with chronic diseases

Table 2 shows the compositional geometric means and variation matrix of 24-h movement behaviors. In terms of time composition, SB accounted for the largest proportion of the 24-h day (geometric mean 720.66 min/day, 51.79%), followed by sleep (485.69 min/day, 34.90%), then LPA (153.71 min/day, 11.05%), with MVPA accounting for the smallest proportion (31.43 min/day, 2.26%). The variation matrix reflects the interdependence among behavior components. The log-ratio variance between sleep and SB was the smallest (0.146), indicating the strongest interdependence and the greatest likelihood of interchange between these two behaviors. In contrast, the log-ratio variance between SB and MVPA was the largest (0.796), indicating the weakest interdependence and the lowest likelihood of interchange.

**TABLE 2:** Time distribution and variance matrix of participants’ 24-h movement behaviors.

Movement behavior	Sleep	SB	LPA	MVPA
Compositional geometric mean (min/day) (%)	485.69 (34.90%)	720.66 (51.79%)	153.71 (11.05%)	31.43 (2.26%)
Sleep	0	0.146	0.220	0.653
SB	0.146	0	0.390	0.796
LPA	0.220	0.390	0	0.607
MVPA	0.653	0.796	0.607	0

### 3.3 Compositional regression analysis of 24-h movement behaviors and psychological distress

Linear regression models based on ILR-transformed coordinates (Table 3) indicated that, after adjustment for confounding factors, specific behavior components were significantly associated with psychological distress (overall adjusted R^2^ = 0.26). Specifically, relative to the geometric mean of the remaining behaviors, a higher proportion of MVPA time was significantly associated with lower psychological distress (*B* = −2.96, *p* = 0.011), indicating that greater MVPA proportion corresponded to lower psychological distress. LPA showed a similar inverse association with psychological distress (*B* = −2.53, *p* < 0.001). In contrast, a higher SB proportion was significantly associated with higher psychological distress (*B* = 5.11, *p* < 0.001), indicating an unfavorable psychological distress profile among participants with relatively greater sedentary time. In this model, the relative proportion of sleep was not significantly associated with psychological distress (*p* = 0.831) (Supplementary Appendix 2).

**TABLE 3:** Association between 24-h movement behaviors and psychological distress.

Behavior	*B*	SE	*p*	R^2^	Adjusted R^2^
ILR/ln (Sleep: geometric mean of remaining behaviors)	0.37	1.74	0.831	0.31	0.26
ILR/ln (SB: geometric mean of remaining behaviors)	5.11	1.34	< 0.001		
ILR/ln (MVPA: geometric mean of remaining behaviors)	−2.96	1.15	0.011		
ILR/ln (LPA: geometric mean of remaining behaviors)	−2.53	0.74	< 0.001		

The model was adjusted for sex, age, BMI, education level, socioeconomic status, number of chronic diseases, and Social Support Rating Scale. *B* indicates unstandardized regression coefficient estimates from the compositional linear regression using ILR-transformed movement behaviors; SE, standard error of unstandardized regression coefficient.

### 3.4 Isotemporal substitution effects of 24-h time reallocation on psychological distress

The 15-min substitution analysis showed three main patterns (Table 4). First, reallocations involving MVPA showed the largest estimated differences in psychological distress. Reallocating 15 min from SB or sleep to MVPA was associated with lower predicted K10 scores, with the largest difference observed for SB→MVPA (−0.92, 95% CI: −1.41 to −0.43), followed by sleep→MVPA (−0.84, 95% CI: −1.34 to −0.34). Conversely, reallocating 15 min away from MVPA to SB, sleep, or LPA was associated with higher predicted K10 scores, with estimates ranging from +1.12 to +1.44; all corresponding 95% CIs excluded zero. Second, reallocations between SB and LPA showed smaller but consistent associations. Reallocating 15 min from SB to LPA was associated with a lower predicted K10 score (−0.32, 95% CI: −0.50 to −0.14), whereas reallocating 15 min from LPA to SB was associated with a higher predicted K10 score (+0.34, 95% CI: 0.14 to 0.54). Similar but smaller associations were observed for sleep→LPA and LPA→sleep. Third, reallocations between sleep and SB were close to null. Reallocating 15 min from sleep to SB or from SB to sleep was associated with only minimal estimated differences in K10, and the corresponding 95% CIs included zero.

**TABLE 4:** Changes in psychological distress associated with 15-min isocaloric substitution of 24-h movement behaviors in older adults with chronic diseases.

Movement behavior	MVPA↑	LPA↑	Sleep↑	SB↑
MVPA↓	—	1.12 (0.27, 1.98)*	1.36 (0.57, 2.16)*	1.44 (0.66, 2.23)*
LPA↓	−0.58 (−1.15, −0.00)*	—	0.26 (0.01, 0.52)*	0.34 (0.14, 0.54)*
Sleep↓	−0.84 (−1.34, −0.34)*	−0.24 (−0.48, −0.00)*	—	0.08 (−0.05, 0.21)
SB↓	−0.92 (−1.41, −0.43)*	−0.32 (−0.50, −0.14)*	−0.08 (−0.21, 0.05)	—

↑indicates a 15-min increase in the duration of the activity behavior; ↓indicates a 15-min decrease in the duration of the activity behavior; *indicates that the 95% CI does not include 0.

### 3.5 Dose–response relationship between 24-h time reallocation and psychological distress

**FIGURE 1:**
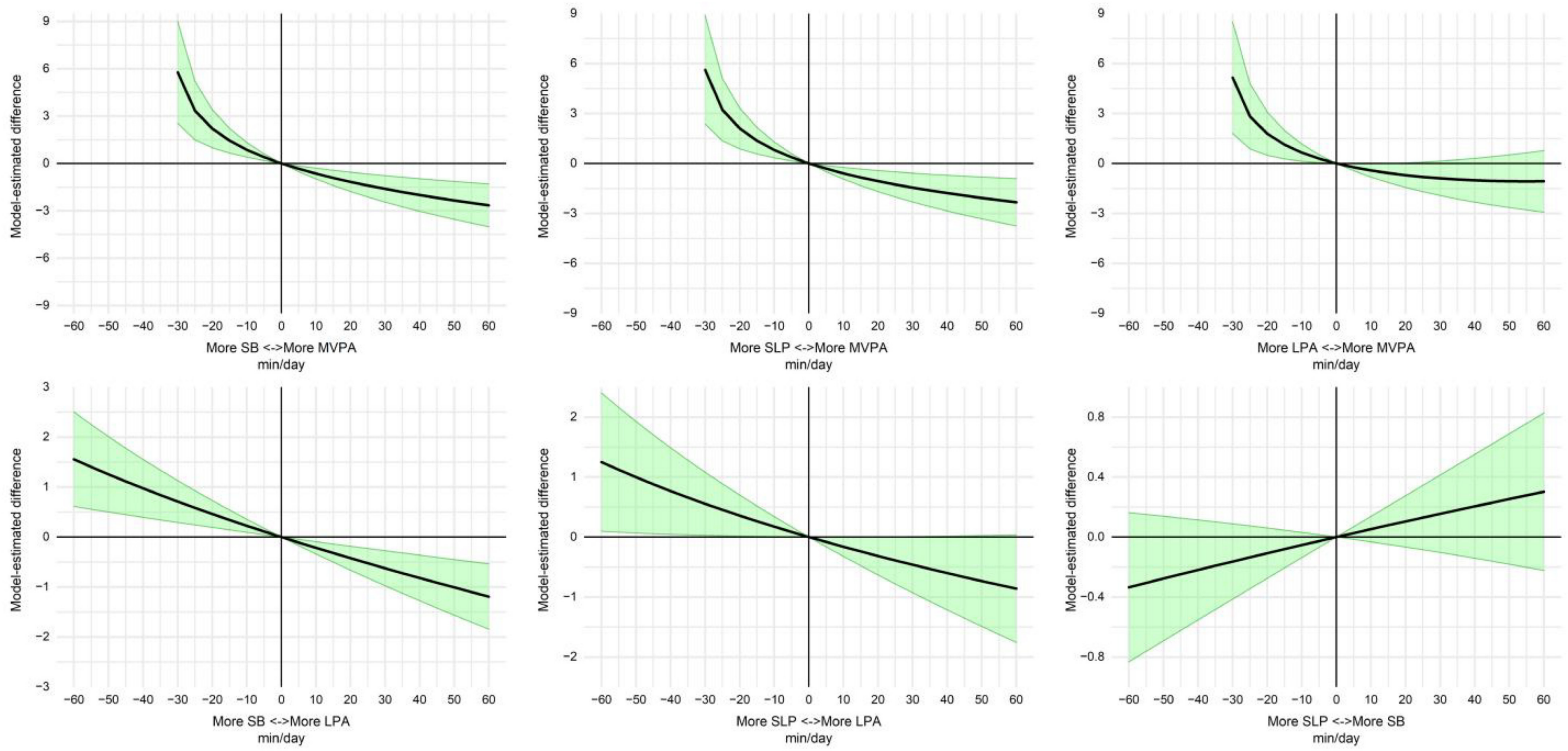
Dose-response relationship between temporal redistribution of 24-h activity behaviors and psychological distress. The horizontal axis represents the number of minutes reallocated from one behavior to another, as indicated in each panel. The vertical axis represents the estimated change in K10 score after reallocating that amount of time, compared with no reallocation between the two behaviors; values below zero indicate lower estimated psychological distress after the reallocation, whereas values above zero indicate higher estimated psychological distress. Confidence intervals crossing zero indicate that the estimated difference was not statistically significant.

The dose–response analysis further supported the pattern observed in the 15-min substitution analysis (Figure 1; Supplementary Appendix 3). The clearest gradients were found for reallocations involving MVPA. When time was progressively reallocated from SB to MVPA, the predicted K10 score became increasingly lower, from −0.34 (95% CI: −0.53 to −0.16) at 5 min to −1.61 (95% CI: −2.45 to −0.77) at 30 min and −2.66 (95% CI: −4.01 to −1.31) at 60 min. A similar pattern was observed for reallocations from sleep to MVPA, with estimates of −0.32 (95% CI: −0.50 to −0.13), −1.45 (95% CI: −2.32 to −0.58), and −2.33 (95% CI: −3.75 to −0.91) at 5, 30, and 60 min, respectively.

In the opposite direction, reallocating time away from MVPA was associated with higher predicted K10 scores. Because MVPA accounted for the smallest proportion of the 24-h composition, estimates for reallocations away from MVPA were interpretable up to 30 min. Within this range, the largest positive estimates were observed when MVPA was reallocated to SB or sleep. For example, at 30 min, the predicted K10 score was higher for MVPA→SB (+5.77, 95% CI: 2.53 to 9.01), MVPA→sleep (+5.61, 95% CI: 2.36 to 8.87), and MVPA→LPA (+5.15, 95% CI: 1.80 to 8.51).

Reallocations between SB and LPA showed more moderate but consistent dose–response patterns. Reallocating time from SB to LPA was associated with progressively lower predicted K10 scores, whereas reallocating time from LPA to SB was associated with progressively higher predicted K10 scores. By contrast, reallocations between sleep and SB remained close to null across all examined doses and were not statistically significant. Taken together, the dose–response results indicate that the most interpretable and clinically relevant patterns were concentrated in substitutions involving MVPA, while replacing SB with LPA showed a smaller but stable association with lower psychological distress.

The results of the sensitivity analysis excluding social support were broadly consistent with the main analysis, indicating that the primary findings were robust (Supplementary Appendix 4).

## 4 Discussion

### 4.1 Main findings

Using compositional isotemporal substitution analysis from a whole-day time-composition perspective, this study examined the associations between 24-h movement behaviors and psychological distress among older adults with chronic diseases. The findings suggest that the overall composition of 24-h movement behaviors is significantly associated with psychological distress. Higher relative proportions of MVPA and LPA were associated with lower psychological distress, whereas a higher proportion of SB was associated with higher psychological distress. In the isotemporal substitution models, reallocations involving MVPA were associated with the largest estimated differences in K10, followed by reallocations involving LPA, whereas substitutions between sleep and SB showed little association. Importantly, this study also adds to the literature by using wrist accelerometry rather than questionnaire-based time-use estimates, which may reduce recall-related misclassification and allow finer quantification of the full 24-h composition (Prince et al., 2020). By focusing on psychological distress as a broader construct that encompasses depressive and anxiety-related symptoms, the present analysis complements prior work that has often focused on single mental health outcomes.

### 4.2 Time-composition characteristics of 24-h movement behaviors in older adults with chronic diseases

The results showed that older adults with chronic diseases spent an average of 720.7 min per day in SB (51.8% of 24 h), whereas MVPA averaged 31.4 min (2.26%). Although the latter exceeds the minimum recommendation by the World Health Organization (WHO) of 150 min of MVPA per week for older adults (i.e., approximately 21.4 min/day) (Bull et al., 2020), its proportion of total 24-h time is very low, and given the large individual variability (as shown in Table 1), it suggests that a considerable proportion of older adults still do not meet or only barely meet recommendations. This aligns with recent findings on adherence to 24-h movement guidelines among Chinese older adults, which also highlight challenges in meeting physical activity targets (Liang et al., 2024a). This pattern may be attributable to pain, fatigue, and functional limitations associated with chronic diseases (Demircioglu-Karagoz et al., 2025), as well as the combined influence of social environmental and psychological factors (Ji et al., 2025).

Previous studies often assessed a single behavior in isolation and overlooked the trade-offs among behaviors (Chastin et al., 2015). In contrast, the variation matrix (Table 2) in the present study provides deeper insight into interdependence among 24-h behaviors. The smallest log-ratio variance was observed between sleep and SB (0.146), indicating that these two static behaviors are most likely to interchange in daily life. This may be because when older adults with chronic diseases feel physically uncomfortable or lack opportunities for activity, their default state is to alternate between “sitting” and “lying down,” suggesting that without external interventions or intrinsic motivation, reduced sedentary time is most likely to be replaced by time spent in bed. These two behaviors thus constitute the “behavioral background” of daily life and form a close alliance within the time budget. In contrast, the largest log-ratio variance between MVPA and SB (0.796) suggests that converting sedentary time into high-intensity activity faces the greatest practical barriers. This quantitatively reflects a common observation in clinical practice: encouraging older adults with chronic diseases to engage in brisk walking or running is extremely challenging and requires overcoming substantial physiological barriers (e.g., joint pain and insufficient cardiopulmonary function) as well as psychological barriers (e.g., lack of confidence and inertia of habits) (Mizutani and Ukawa, 2025). Therefore, especially in the early stages of intervention, strategy design should fully consider the feasibility of behavioral transitions; for example, promoting the shift from SB to LPA may be more cost-effective and practically feasible than focusing solely on MVPA.

### 4.3 Relationship between 24-h movement behaviors and psychological distress in older adults with chronic diseases

Using the compositional regression model, we observed clear associations between the 24-h movement behavior composition and psychological distress. Consistent with established evidence, a greater proportion of MVPA was inversely associated with psychological distress scores (*B* = −2.96, SE = 1.15) (Mizutani and Ukawa, 2025). This inverse relationship was reinforced by the isotemporal substitution models, where reallocating time to MVPA from any other behavior yielded the largest estimated reductions in K10 scores. This finding aligns with established neurobiological pathways, such as the release of endorphins and serotonin (Kostka et al., 2024), and psychosocial benefits, including enhanced self-efficacy and social interaction (Ji et al., 2025). Critically, the isotemporal substitution models quantified this relationship, showing that reallocating time to MVPA yielded the largest estimated reductions in K10 scores. The models also revealed a notable “asymmetry”: the adverse association linked to losing MVPA was numerically larger than the favorable association linked to an equivalent gain. For instance, reallocating 15 min of MVPA to SB was associated with a 1.44-point increase in K10, whereas the reverse reallocation was associated with a smaller 0.92-point decrease. This suggests that for this vulnerable population, preserving existing MVPA may be as important as promoting new activity.

Notably, a higher proportion of LPA was also independently associated with lower psychological distress (*B* = −2.53, *p* < 0.001). This is a critical finding, as LPA has historically been overlooked in favor of MVPA, often considered to have limited health relevance (Piercy et al., 2018). Our results demonstrate that even after accounting for the contributions of other behaviors, LPA remains a significant correlate of better mental wellbeing. The isotemporal substitution analyses further supported this, showing that reallocations involving LPA were linked to moderate, yet potentially meaningful, reductions in distress. This contributes to a growing body of evidence that LPA confers independent mental health benefits (Teno et al., 2024). For older adults with chronic diseases, whose capacity for vigorous activity may be limited, this finding is particularly salient. It suggests that incorporating more low-intensity activities such as walking and household chores represents a feasible and effective strategy to improve psychological status. From a practical standpoint, this underscores LPA’s value as an accessible, low-barrier entry point for reducing SB.

In contrast to the beneficial associations observed for physical activity, SB and sleep exhibited divergent relationships with psychological distress. A higher proportion of SB was strongly associated with greater psychological distress (*B* = 5.11, *p* < 0.001), whereas the relative proportion of sleep showed no significant association (*p* = 0.831). The null finding for sleep may seem at odds with the extensive literature linking sleep problems to depression and anxiety (Yin et al., 2017). However, this should not be interpreted as sleep being unimportant. Instead, it suggests that within the statistical constraints of a 24-h composition, the relative duration of sleep was not an independent predictor of distress in this cohort. Several factors may explain this result. First, as a compositional variable, an increase in sleep’s proportion necessitates a decrease elsewhere. If additional sleep time primarily displaces MVPA or LPA, any potential benefit of longer sleep could be neutralized by the detrimental effect of reduced physical activity. The isotemporal substitution results support this, showing that reallocating time from active behaviors to sleep was associated with higher estimated K10 scores. Second, the mean sleep duration in our sample (∼8.2 h) was already near the upper limit recommended for older adults (7–8 h) (Koffel et al., 2023). Given the often U-shaped relationship between sleep duration and health, our participants may have been operating outside the range where additional sleep time is beneficial (Mogavero et al., 2025). Finally, and perhaps most critically, this finding may reflect a measurement limitation. Our use of actigraphy, while objective, captures sleep duration based on inactivity and cannot assess the neurophysiological architecture or qualitative aspects of sleep (e.g., sleep fragmentation, continuity). The literature suggests that the association between sleep and psychological distress is often more strongly linked to these qualitative metrics than to duration alone. Consequently, our reliance on a purely quantitative measure may have obscured an underlying association. The non-significant result may therefore indicate the insensitivity of sleep duration as a standalone metric in this context, rather than a true absence of a relationship. Future studies incorporating polysomnography or validated multi-sensor sleep technologies are warranted to delineate the role of sleep quality.

### 4.4 Associations of isotemporal substitution of 24-h movement behaviors on psychological distress

Another contribution of this study is the quantification of estimated differences in K10 associated with specific reallocations of 24-h time. Reallocations toward MVPA were associated with the largest favorable differences in psychological distress. Specifically, when 15 min were reallocated to MVPA from sedentary time or sleep, the model estimated lower K10 values (−0.92 and −0.84 points, respectively). These findings provide an empirical basis for prioritizing movement-behavior targets in future longitudinal or intervention research, rather than evidence of direct intervention effects in the present cross-sectional study.

More importantly, the model suggested an “asymmetry” and a “priority ranking” in reallocations across 24-h movement behaviors. The adverse association linked to lower MVPA was numerically larger than the favorable association linked to an equivalent MVPA gain. When 15 min of MVPA was reallocated to SB, K10 was estimated to be 1.44 points higher, whereas the reverse reallocation was associated with a 0.92-point lower K10. A similar asymmetry was also reflected in the dose-response curves: the gradient associated with reallocations away from MVPA was steeper than the gradient associated with reallocations toward MVPA. Although these patterns should not be interpreted causally, they suggest that lower MVPA is closely linked to poorer psychological distress profiles in this sample. For older adults with chronic diseases who already accumulate relatively little MVPA, preserving existing activity may therefore be an especially relevant consideration for future intervention design.

Furthermore, the clinical implications of our findings extend beyond MVPA. Although reallocations to LPA yielded smaller effect sizes than those to MVPA, the practical accessibility of LPA makes it a highly relevant intervention target, particularly for this population. The observation that reallocating just 15 min from SB to LPA was associated with a statistically significant decrease in psychological distress (−0.34 points on the K10 scale) is noteworthy. Given that transitions from SB to LPA are considerably more attainable for older adults with functional limitations than transitions to MVPA, this finding provides an empirical basis for designing stepwise behavioral interventions (Petrusevski et al., 2021). From a clinical perspective, generic advice such as “exercise more” can be translated into specific, behavior-focused recommendations. For instance, suggesting the replacement of short periods of sitting with light ambulation or standing breaks is a tangible first step. To enhance the practical utility of these findings, interventions could be structured around two primary goals:

1. Promoting LPA and reducing SB: This strategy focuses on integrating low-intensity activities into daily routines. Examples include incorporating stretching breaks after every 30 min of sitting, engaging in a 10-min walk post-meal, or performing light household chores for 15–20 min. Participation in gentle, structured activities like Tai Chi or Qigong, adapted for individuals with chronic conditions, could also be encouraged.
2. Gradual integration of MVPA: For individuals capable of more intense activity, a progressive approach is recommended. This could involve initiating 5–10 min of moderate-intensity exercise (e.g., brisk walking, stationary cycling) 2–3 times per week, with duration increased incrementally (e.g., by 5 min every two weeks) as tolerated. Intensity should be monitored based on individual capacity, such as maintaining a heart rate within 60%–70% of the age-predicted maximum and avoiding undue fatigue or pain. For those with severe conditions, such as advanced cardiopulmonary disease, any engagement in MVPA must be conducted under medical or physical therapy supervision to ensure safety.

Finally, reallocations between SB and sleep were not significantly associated with psychological distress, suggesting that, in this sample, shifting time between two relatively inactive behaviors was not linked to meaningful differences in K10. When sleep decreased by 15 min and was reallocated to SB, the estimated K10 change was only +0.08 (95% CI: −0.05, 0.21), whereas the reverse reallocation yielded −0.08 (95% CI: −0.21, 0.05); neither was significant. This pattern is particularly relevant for older adults who tend to alternate between the sofa and the bed because of pain or fatigue, as it suggests that simply shifting time between two inactive states may not be associated with better psychological distress profiles. A plausible explanation is that both SB and sleep involve little muscular activity and limited behavioral engagement, whereas even low-intensity movement may provide a greater degree of physiological activation and behavioral interruption (Bergouignan et al., 2016; Owen et al., 2010).

### 4.5 Limitations

While this study is innovative in methodology and perspective, its inherent limitations also indicate directions for future research. First, the present study was a restricted analysis based on a broader community sample. Because only older adults with chronic diseases were included in the final analysis, potential selection bias cannot be completely excluded, and the generalizability of the findings to the overall older adult population may be limited. In addition, no participant in the final analytic sample fell into the Q4 range of the K10 (30–50). This pattern likely reflects the community-based, relatively functional nature of the sample and the study requirements for completing questionnaires and valid accelerometer wear, rather than an explicit exclusion based on psychological distress. Therefore, our findings may be less generalizable to older adults with very severe psychological distress. Beyond this, residual clinical confounding may still remain in older adults with chronic diseases, particularly from factors such as pain, functional limitations, and disease severity (Mizutani and Ukawa, 2025; Palmese et al., 2025). Future studies should further account for these key clinical characteristics to provide more robust evidence on the association between 24-h movement behaviors and psychological distress in this population. Second, due to the cross-sectional design, only associations can be observed, and causality cannot be established. The temporal sequence between 24-h movement behaviors and psychological distress remains unclear, and the observed associations may be bidirectional. For example, less favorable movement patterns may be associated with higher psychological distress, while psychological distress may also reduce motivation, energy, and social participation, thereby contributing to less active daily behaviors. Future studies should adopt longitudinal cohort designs with repeated measurements, cross-lagged panel models, or intervention and natural-experimental designs to clarify temporal precedence and provide stronger evidence regarding causal pathways. Third, objective accelerometer measures still have limitations. As noted, they cannot distinguish sleep quality and cannot identify the specific context of SB (e.g., reading, socializing, or watching TV), and different types of SB may have markedly different effects on mental health. To fill this methodological gap, future studies may combine logs or ecological momentary assessment (EMA) to further differentiate SB types, providing accurate scientific evidence for developing 24-h activity guidelines for older adults with chronic diseases in China.

## 5 Conclusion

In older adults with chronic diseases, the 24-h composition of movement behaviors is significantly linked to their psychological distress. Our findings reveal a clear hierarchy, higher proportions of MVPA and LPA were associated with better psychological outcomes, whereas more sedentary time was linked to greater distress. Notably, preserving existing MVPA proved highly impactful, while substitutions between inactive states (sleep and sedentary time) offered little benefit. A key takeaway for practice is that clinicians can integrate specific LPA goals into chronic disease management, while public policy should focus on building supportive communities through tailored programs and accessible urban design. The next step is to rigorously test these interventions. Such research is essential to move beyond associations, establish causality, and ultimately develop new 24-h activity guidelines that are truly tailored to the needs and capacities of this population.

## Data Availability

The raw data supporting the conclusions of this article will be made available by the authors, without undue reservation.
