# Supplementary material for: Association between 24-h movement behaviors and psychological distress in older adults with chronic diseases: a compositional isotemporal substitution analysis
Source: Front Psychol. 2026 May 19;17:1782087. doi: 10.3389/fpsyg.2026.1782087 (PMC13226525; doi:10.3389/fpsyg.2026.1782087)
Supplement: Supplementary file 1 [file Supplementary_file_1.docx]

**Appendix 1. Methodological details for the ilr basis, sequential binary partition, and inference for compositional isotemporal substitution**

# **Appendix 1.1 Analytical overview**

Two related analytical layers were used in the study. First, for prediction-based compositional isotemporal substitution, the four 24-h movement behavior components were represented in an orthonormal ilr coordinate system and entered into the fitted linear regression model to obtain predicted K10 values for the reference and reallocated compositions. Second, for behavior-specific interpretation, pivot-coordinate parameterizations were fitted separately for sleep, SB, LPA, and MVPA. In each pivot-coordinate model, the first ilr coordinate represented the focal behavior relative to the geometric mean of the remaining three behaviors.

Accordingly, p-values reported for individual movement behaviors were obtained from the coefficient test of the first pivot-coordinate ilr term in the corresponding regression model, whereas statistical inference for isotemporal substitution estimates was based on the 95% confidence intervals of the predicted differences.

# **Appendix 1.2 Coordinate definitions**

Let the 24-h movement behavior composition be x = (x_sleep, x_SB, x_LPA, x_MVPA), where all parts are positive and sum to 1440 min/day. For a 4-part composition (D = 4), the first pivot coordinate for a focal behavior was defined as:

z1 = sqrt((D−1)/D) × ln[x_focal / (x_a x_b x_c)^(1/(D−1))], D = 4

where x_a, x_b, and x_c denote the remaining three behaviors. Thus, z1 captures the relative information for the focal behavior against the geometric mean of the remaining parts. The remaining two pivot coordinates were generated from the nested SBP structures shown below.

# **Appendix 1.3 Sequential binary partition (SBP) sign matrices used for behavior-specific pivot coordinates**

Columns are ordered as Sleep, SB, LPA, and MVPA. Each row corresponds to one ilr coordinate. The first row is the focal behavior versus the remaining three behaviors.

## **Sleep-focused pivot coordinates**

|  | Sleep | SB | LPA | MVPA |
| --- | --- | --- | --- | --- |
| ilr1 | 1 | -1 | -1 | -1 |
| ilr2 | 0 | 1 | -1 | -1 |
| ilr3 | 0 | 0 | 1 | -1 |

*For the sleep-focused model, the first coordinate (ilr1) represents sleep relative to the geometric mean of the other three behaviors.*

## **SB-focused pivot coordinates**

|  | Sleep | SB | LPA | MVPA |
| --- | --- | --- | --- | --- |
| ilr1 | -1 | 1 | -1 | -1 |
| ilr2 | 1 | 0 | -1 | -1 |
| ilr3 | 0 | 0 | 1 | -1 |

*For the sb-focused model, the first coordinate (ilr1) represents sb relative to the geometric mean of the other three behaviors.*

## **LPA-focused pivot coordinates**

|  | Sleep | SB | LPA | MVPA |
| --- | --- | --- | --- | --- |
| ilr1 | -1 | -1 | 1 | -1 |
| ilr2 | 1 | -1 | 0 | -1 |
| ilr3 | 0 | 1 | 0 | -1 |

*For the lpa-focused model, the first coordinate (ilr1) represents lpa relative to the geometric mean of the other three behaviors.*

## **MVPA-focused pivot coordinates**

|  | Sleep | SB | LPA | MVPA |
| --- | --- | --- | --- | --- |
| ilr1 | -1 | -1 | -1 | 1 |
| ilr2 | 1 | -1 | -1 | 0 |
| ilr3 | 0 | 1 | -1 | 0 |

*For the mvpa-focused model, the first coordinate (ilr1) represents mvpa relative to the geometric mean of the other three behaviors.*

# **Appendix 1.4 Prediction-based compositional isotemporal substitution**

Let x_ref denote the reference composition and x_new denote a reallocated composition created by transferring a fixed amount of time from one behavior to another while preserving the 24-h total. After transforming each composition into the same ilr basis used in the fitted prediction model, the expected isotemporal substitution effect was defined as the difference between the two model-predicted K10 values:

Δ = ŷ(x_new) − ŷ(x_ref) = (X_new − X_ref)^T β̂

Here, X denotes the full model design vector, including the ilr coordinates and the covariates. Because covariates were held constant across prediction scenarios, only the ilr-coordinate elements differed between X_new and X_ref.

# **Appendix 1.5 Standard error, confidence interval, and significance rule for substitution estimates**

The standard error of the predicted difference was computed as the standard error of a linear contrast of the regression coefficients:

SE(Δ) = sqrt[(X_new − X_ref)^T Var(β̂) (X_new − X_ref)]

95% CI = Δ ± t0.975,df × SE(Δ)

An isotemporal substitution estimate was regarded as statistically significant when its 95% confidence interval did not include zero. This is equivalent to a two-sided Wald t test for the same linear contrast.

# **Appendix 1.6 Reproducible R code template**

The analytical code can be referenced following the methods provided in the link below:

https://www.intue.org/wp-content/uploads/2022/06/Example-CoDA-code-2022.html

**Appendix 2. Association between 24-hour Movement Behaviors and Psychological Distress**

| Behavior | *β* | *β*_95CI_low | *β*_95CI_high | *SE*_std | *p* |
| --- | --- | --- | --- | --- | --- |
| ILR/ln (Sleep: geometric mean of remaining behaviors) | 0.02 | -0.17 | 0.21 | 0.10 | .831 |
| ILR/ln (SB: geometric mean of remaining behaviors) | 0.41 | 0.20 | 0.62 | 0.11 | < .001 |
| ILR/ln (MVPA: geometric mean of remaining behaviors) | -0.21 | -0.38 | -0.05 | 0.08 | .011 |
| ILR/ln (LPA: geometric mean of remaining behaviors) | -0.29 | -0.46 | -0.12 | 0.09 | < .001 |
| The model was adjusted for sex, age, BMI, education level, socioeconomic status, number of chronic diseases, and Social Support Rating Scale. *β* indicates standardized regression coefficient estimates from the compositional linear regression using ilr-transformed movement behaviors; *SE*_std, Standard Error of Standardized Regression Coefficient. | | | | | |

**Appendix 3. Dose–Response Relationship Between 24-h Time Reallocation and Psychological Distress**

| increase  behavior | decrease  behavior | delta_min | Effect (*B*)_ci ^1^ | effect_sd_ci ^1^ | *p*_value ^1^ |
| --- | --- | --- | --- | --- | --- |
| LPA | MVPA | 5 | 0.29 (0.05, 0.52)* | 0.05 (0.01, 0.09)* | 0.019 |
| LPA | MVPA | 10 | 0.65 (0.13, 1.16)* | 0.11 (0.02, 0.20)* | 0.014 |
| LPA | MVPA | 15 | 1.12 (0.27, 1.98)* | 0.19 (0.05, 0.34)* | 0.011 |
| LPA | MVPA | 20 | 1.79 (0.49, 3.08)** | 0.31 (0.08, 0.54)** | 0.008 |
| LPA | MVPA | 25 | 2.83 (0.87, 4.79)** | 0.49 (0.15, 0.83)** | 0.005 |
| LPA | MVPA | 30 | 5.15 (1.80, 8.51)** | 0.89 (0.31, 1.48)** | 0.003 |
| LPA | SB | 5 | -0.11 (-0.17, -0.05)** | -0.02 (-0.03, -0.01)** | 0.001 |
| LPA | SB | 10 | -0.22 (-0.34, -0.09)** | -0.04 (-0.06, -0.02)** | 0.001 |
| LPA | SB | 15 | -0.32 (-0.50, -0.14)** | -0.06 (-0.09, -0.02)** | 0.001 |
| LPA | SB | 20 | -0.42 (-0.66, -0.18)** | -0.07 (-0.12, -0.03)** | 0.001 |
| LPA | SB | 25 | -0.53 (-0.82, -0.23)** | -0.09 (-0.14, -0.04)** | 0.001 |
| LPA | SB | 30 | -0.62 (-0.98, -0.27)** | -0.11 (-0.17, -0.05)** | 0.001 |
| LPA | SB | 35 | -0.72 (-1.13, -0.32)** | -0.13 (-0.20, -0.06)** | 0.001 |
| LPA | SB | 40 | -0.82 (-1.28, -0.36)** | -0.14 (-0.22, -0.06)** | 0.001 |
| LPA | SB | 45 | -0.91 (-1.42, -0.41)** | -0.16 (-0.25, -0.07)** | 0.001 |
| LPA | SB | 50 | -1.01 (-1.57, -0.45)** | -0.17 (-0.27, -0.08)** | 0.001 |
| LPA | SB | 55 | -1.10 (-1.71, -0.49)** | -0.19 (-0.30, -0.09)** | 0.001 |
| LPA | SB | 60 | -1.19 (-1.85, -0.54)** | -0.21 (-0.32, -0.09)** | < .001 |
| LPA | SLP | 5 | -0.08 (-0.16, -0.00)* | -0.01 (-0.03, -0.00)* | 0.047 |
| LPA | SLP | 10 | -0.16 (-0.32, -0.00)* | -0.03 (-0.06, -0.00)* | 0.048 |
| LPA | SLP | 15 | -0.24 (-0.48, -0.00)* | -0.04 (-0.08, -0.00)* | 0.049 |
| LPA | SLP | 20 | -0.32 (-0.63, 0.00) | -0.05 (-0.11, 0.00) | 0.05 |
| LPA | SLP | 25 | -0.39 (-0.78, 0.00) | -0.07 (-0.14, 0.00) | 0.051 |
| LPA | SLP | 30 | -0.46 (-0.93, 0.00) | -0.08 (-0.16, 0.00) | 0.052 |
| LPA | SLP | 35 | -0.53 (-1.07, 0.01) | -0.09 (-0.19, 0.00) | 0.054 |
| LPA | SLP | 40 | -0.60 (-1.22, 0.01) | -0.10 (-0.21, 0.00) | 0.055 |
| LPA | SLP | 45 | -0.67 (-1.35, 0.02) | -0.12 (-0.23, 0.00) | 0.056 |
| LPA | SLP | 50 | -0.73 (-1.49, 0.02) | -0.13 (-0.26, 0.00) | 0.057 |
| LPA | SLP | 55 | -0.80 (-1.63, 0.03) | -0.14 (-0.28, 0.01) | 0.059 |
| LPA | SLP | 60 | -0.86 (-1.76, 0.04) | -0.15 (-0.31, 0.01) | 0.06 |
| MVPA | LPA | 5 | -0.23 (-0.44, -0.02)* | -0.04 (-0.08, -0.00)* | 0.031 |
| MVPA | LPA | 10 | -0.42 (-0.82, -0.02)* | -0.07 (-0.14, -0.00)* | 0.039 |
| MVPA | LPA | 15 | -0.58 (-1.15, -0.00)* | -0.10 (-0.20, -0.00)* | 0.049 |
| MVPA | LPA | 20 | -0.70 (-1.44, 0.03) | -0.12 (-0.25, 0.01) | 0.06 |
| MVPA | LPA | 25 | -0.81 (-1.70, 0.08) | -0.14 (-0.29, 0.01) | 0.074 |
| MVPA | LPA | 30 | -0.89 (-1.93, 0.14) | -0.16 (-0.33, 0.02) | 0.09 |
| MVPA | LPA | 35 | -0.96 (-2.14, 0.22) | -0.17 (-0.37, 0.04) | 0.109 |
| MVPA | LPA | 40 | -1.01 (-2.33, 0.30) | -0.18 (-0.40, 0.05) | 0.13 |
| MVPA | LPA | 45 | -1.05 (-2.50, 0.40) | -0.18 (-0.43, 0.07) | 0.155 |
| MVPA | LPA | 50 | -1.07 (-2.65, 0.51) | -0.19 (-0.46, 0.09) | 0.183 |
| MVPA | LPA | 55 | -1.08 (-2.80, 0.64) | -0.19 (-0.48, 0.11) | 0.216 |
| MVPA | LPA | 60 | -1.07 (-2.92, 0.77) | -0.19 (-0.51, 0.13) | 0.253 |
| MVPA | SB | 5 | -0.34 (-0.53, -0.16)** | -0.06 (-0.09, -0.03)** | < .001 |
| MVPA | SB | 10 | -0.65 (-0.99, -0.30)** | -0.11 (-0.17, -0.05)** | < .001 |
| MVPA | SB | 15 | -0.92 (-1.41, -0.43)** | -0.16 (-0.24, -0.08)** | < .001 |
| MVPA | SB | 20 | -1.17 (-1.78, -0.56)** | -0.20 (-0.31, -0.10)** | < .001 |
| MVPA | SB | 25 | -1.40 (-2.13, -0.67)** | -0.24 (-0.37, -0.12)** | < .001 |
| MVPA | SB | 30 | -1.61 (-2.45, -0.77)** | -0.28 (-0.43, -0.13)** | < .001 |
| MVPA | SB | 35 | -1.81 (-2.75, -0.87)** | -0.31 (-0.48, -0.15)** | < .001 |
| MVPA | SB | 40 | -2.00 (-3.03, -0.97)** | -0.35 (-0.53, -0.17)** | < .001 |
| MVPA | SB | 45 | -2.18 (-3.30, -1.06)** | -0.38 (-0.57, -0.18)** | < .001 |
| MVPA | SB | 50 | -2.35 (-3.55, -1.14)** | -0.41 (-0.62, -0.20)** | < .001 |
| MVPA | SB | 55 | -2.51 (-3.78, -1.23)** | -0.43 (-0.66, -0.21)** | < .001 |
| MVPA | SB | 60 | -2.66 (-4.01, -1.31)** | -0.46 (-0.70, -0.23)** | < .001 |
| MVPA | SLP | 5 | -0.32 (-0.50, -0.13)** | -0.05 (-0.09, -0.02)** | 0.001 |
| MVPA | SLP | 10 | -0.59 (-0.94, -0.24)** | -0.10 (-0.16, -0.04)** | 0.001 |
| MVPA | SLP | 15 | -0.84 (-1.34, -0.34)** | -0.15 (-0.23, -0.06)** | 0.001 |
| MVPA | SLP | 20 | -1.06 (-1.69, -0.43)** | -0.18 (-0.29, -0.07)** | 0.001 |
| MVPA | SLP | 25 | -1.26 (-2.02, -0.51)** | -0.22 (-0.35, -0.09)** | 0.001 |
| MVPA | SLP | 30 | -1.45 (-2.32, -0.58)** | -0.25 (-0.40, -0.10)** | 0.001 |
| MVPA | SLP | 35 | -1.62 (-2.60, -0.65)** | -0.28 (-0.45, -0.11)** | 0.001 |
| MVPA | SLP | 40 | -1.78 (-2.86, -0.71)** | -0.31 (-0.50, -0.12)** | 0.001 |
| MVPA | SLP | 45 | -1.93 (-3.10, -0.76)** | -0.34 (-0.54, -0.13)** | 0.001 |
| MVPA | SLP | 50 | -2.07 (-3.33, -0.82)** | -0.36 (-0.58, -0.14)** | 0.001 |
| MVPA | SLP | 55 | -2.20 (-3.54, -0.86)** | -0.38 (-0.61, -0.15)** | 0.001 |
| MVPA | SLP | 60 | -2.33 (-3.75, -0.91)** | -0.40 (-0.65, -0.16)** | 0.002 |
| SB | LPA | 5 | 0.11 (0.05, 0.18)** | 0.02 (0.01, 0.03)** | 0.001 |
| SB | LPA | 10 | 0.23 (0.09, 0.36)** | 0.04 (0.02, 0.06)** | 0.001 |
| SB | LPA | 15 | 0.34 (0.14, 0.54)** | 0.06 (0.02, 0.09)** | 0.001 |
| SB | LPA | 20 | 0.46 (0.19, 0.73)** | 0.08 (0.03, 0.13)** | 0.001 |
| SB | LPA | 25 | 0.58 (0.24, 0.93)** | 0.10 (0.04, 0.16)** | 0.001 |
| SB | LPA | 30 | 0.71 (0.29, 1.13)** | 0.12 (0.05, 0.20)** | 0.001 |
| SB | LPA | 35 | 0.84 (0.34, 1.34)** | 0.15 (0.06, 0.23)** | 0.001 |
| SB | LPA | 40 | 0.97 (0.39, 1.55)** | 0.17 (0.07, 0.27)** | 0.001 |
| SB | LPA | 45 | 1.11 (0.45, 1.78)** | 0.19 (0.08, 0.31)** | 0.001 |
| SB | LPA | 50 | 1.25 (0.50, 2.01)** | 0.22 (0.09, 0.35)** | 0.001 |
| SB | LPA | 55 | 1.40 (0.55, 2.25)** | 0.24 (0.10, 0.39)** | 0.001 |
| SB | LPA | 60 | 1.56 (0.61, 2.50)** | 0.27 (0.11, 0.43)** | 0.001 |
| SB | MVPA | 5 | 0.39 (0.18, 0.61)** | 0.07 (0.03, 0.11)** | < .001 |
| SB | MVPA | 10 | 0.86 (0.40, 1.33)** | 0.15 (0.07, 0.23)** | < .001 |
| SB | MVPA | 15 | 1.44 (0.66, 2.23)** | 0.25 (0.11, 0.39)** | < .001 |
| SB | MVPA | 20 | 2.21 (0.99, 3.42)** | 0.38 (0.17, 0.59)** | < .001 |
| SB | MVPA | 25 | 3.35 (1.49, 5.21)** | 0.58 (0.26, 0.90)** | 0.001 |
| SB | MVPA | 30 | 5.77 (2.53, 9.01)** | 1.00 (0.44, 1.56)** | 0.001 |
| SB | SLP | 5 | 0.03 (-0.02, 0.07) | 0.00 (-0.00, 0.01) | 0.222 |
| SB | SLP | 10 | 0.05 (-0.03, 0.14) | 0.01 (-0.01, 0.02) | 0.225 |
| SB | SLP | 15 | 0.08 (-0.05, 0.21) | 0.01 (-0.01, 0.04) | 0.228 |
| SB | SLP | 20 | 0.10 (-0.07, 0.28) | 0.02 (-0.01, 0.05) | 0.231 |
| SB | SLP | 25 | 0.13 (-0.08, 0.34) | 0.02 (-0.01, 0.06) | 0.234 |
| SB | SLP | 30 | 0.15 (-0.10, 0.41) | 0.03 (-0.02, 0.07) | 0.238 |
| SB | SLP | 35 | 0.18 (-0.12, 0.48) | 0.03 (-0.02, 0.08) | 0.241 |
| SB | SLP | 40 | 0.20 (-0.14, 0.55) | 0.04 (-0.02, 0.10) | 0.244 |
| SB | SLP | 45 | 0.23 (-0.16, 0.62) | 0.04 (-0.03, 0.11) | 0.247 |
| SB | SLP | 50 | 0.25 (-0.18, 0.69) | 0.04 (-0.03, 0.12) | 0.251 |
| SB | SLP | 55 | 0.28 (-0.20, 0.76) | 0.05 (-0.04, 0.13) | 0.254 |
| SB | SLP | 60 | 0.30 (-0.22, 0.83) | 0.05 (-0.04, 0.14) | 0.258 |
| SLP | LPA | 5 | 0.09 (0.00, 0.17)* | 0.01 (0.00, 0.03)* | 0.045 |
| SLP | LPA | 10 | 0.17 (0.01, 0.34)* | 0.03 (0.00, 0.06)* | 0.044 |
| SLP | LPA | 15 | 0.26 (0.01, 0.52)* | 0.05 (0.00, 0.09)* | 0.043 |
| SLP | LPA | 20 | 0.36 (0.01, 0.70)* | 0.06 (0.00, 0.12)* | 0.042 |
| SLP | LPA | 25 | 0.45 (0.02, 0.89)* | 0.08 (0.00, 0.15)* | 0.041 |
| SLP | LPA | 30 | 0.55 (0.03, 1.08)* | 0.10 (0.00, 0.19)* | 0.04 |
| SLP | LPA | 35 | 0.66 (0.04, 1.28)* | 0.11 (0.01, 0.22)* | 0.039 |
| SLP | LPA | 40 | 0.77 (0.04, 1.49)* | 0.13 (0.01, 0.26)* | 0.038 |
| SLP | LPA | 45 | 0.88 (0.06, 1.70)* | 0.15 (0.01, 0.30)* | 0.037 |
| SLP | LPA | 50 | 1.00 (0.07, 1.93)* | 0.17 (0.01, 0.33)* | 0.036 |
| SLP | LPA | 55 | 1.12 (0.08, 2.16)* | 0.19 (0.01, 0.37)* | 0.035 |
| SLP | LPA | 60 | 1.25 (0.10, 2.40)* | 0.22 (0.02, 0.42)* | 0.034 |
| SLP | MVPA | 5 | 0.37 (0.15, 0.59)** | 0.06 (0.03, 0.10)** | 0.001 |
| SLP | MVPA | 10 | 0.81 (0.34, 1.29)** | 0.14 (0.06, 0.22)** | 0.001 |
| SLP | MVPA | 15 | 1.36 (0.57, 2.16)** | 0.24 (0.10, 0.37)** | 0.001 |
| SLP | MVPA | 20 | 2.10 (0.88, 3.33)** | 0.36 (0.15, 0.58)** | 0.001 |
| SLP | MVPA | 25 | 3.22 (1.35, 5.09)** | 0.56 (0.23, 0.88)** | 0.001 |
| SLP | MVPA | 30 | 5.61 (2.36, 8.87)** | 0.97 (0.41, 1.54)** | 0.001 |
| SLP | SB | 5 | -0.03 (-0.07, 0.02) | -0.00 (-0.01, 0.00) | 0.216 |
| SLP | SB | 10 | -0.05 (-0.14, 0.03) | -0.01 (-0.02, 0.01) | 0.213 |
| SLP | SB | 15 | -0.08 (-0.21, 0.05) | -0.01 (-0.04, 0.01) | 0.21 |
| SLP | SB | 20 | -0.11 (-0.28, 0.06) | -0.02 (-0.05, 0.01) | 0.207 |
| SLP | SB | 25 | -0.14 (-0.35, 0.07) | -0.02 (-0.06, 0.01) | 0.204 |
| SLP | SB | 30 | -0.16 (-0.41, 0.09) | -0.03 (-0.07, 0.02) | 0.201 |
| SLP | SB | 35 | -0.19 (-0.48, 0.10) | -0.03 (-0.08, 0.02) | 0.198 |
| SLP | SB | 40 | -0.22 (-0.55, 0.11) | -0.04 (-0.10, 0.02) | 0.195 |
| SLP | SB | 45 | -0.25 (-0.62, 0.13) | -0.04 (-0.11, 0.02) | 0.193 |
| SLP | SB | 50 | -0.28 (-0.69, 0.14) | -0.05 (-0.12, 0.02) | 0.190 |
| SLP | SB | 55 | -0.31 (-0.76, 0.15) | -0.05 (-0.13, 0.03) | 0.187 |
| SLP | SB | 60 | -0.34 (-0.83, 0.16) | -0.06 (-0.14, 0.03) | 0.184 |
| ^1^ The model was adjusted for sex, age, BMI, education level, socioeconomic status ,number of chronic diseases, and Social Support Rating Scale; sd: Standard Deviation; * *p*<0.05, ** *p*<0.01 | | | | | |

**Appendix 4. Sensitivity analysis**

| Increase  behavior | Decrease  behavior | delta_min | effect_ci ^2^ | effect_sd_ci ^2^ | *p*_value ^2^ |
| --- | --- | --- | --- | --- | --- |
| LPA | MVPA | 5 | 0.29 (0.06, 0.52)* | 0.05 (0.01, 0.09)* | 0.019 |
| LPA | MVPA | 10 | 0.66 (0.16, 1.16)* | 0.11 (0.03, 0.20)* | 0.014 |
| LPA | MVPA | 15 | 1.14 (0.31, 1.97)** | 0.20 (0.05, 0.34)** | 0.011 |
| LPA | MVPA | 20 | 1.81 (0.55, 3.07)** | 0.31 (0.10, 0.53)** | 0.008 |
| LPA | MVPA | 25 | 2.86 (0.97, 4.76)** | 0.50 (0.17, 0.83)** | 0.005 |
| LPA | MVPA | 30 | 5.21 (1.98, 8.45)** | 0.90 (0.34, 1.47)** | 0.003 |
| LPA | SB | 5 | -0.11 (-0.17, -0.05)** | -0.02 (-0.03, -0.01)** | 0.001 |
| LPA | SB | 10 | -0.22 (-0.34, -0.09)** | -0.04 (-0.06, -0.02)** | 0.001 |
| LPA | SB | 15 | -0.32 (-0.50, -0.14)** | -0.06 (-0.09, -0.02)** | 0.001 |
| LPA | SB | 20 | -0.42 (-0.66, -0.19)** | -0.07 (-0.12, -0.03)** | 0.001 |
| LPA | SB | 25 | -0.53 (-0.82, -0.23)** | -0.09 (-0.14, -0.04)** | 0.001 |
| LPA | SB | 30 | -0.63 (-0.97, -0.28)** | -0.11 (-0.17, -0.05)** | 0.001 |
| LPA | SB | 35 | -0.72 (-1.13, -0.32)** | -0.13 (-0.20, -0.06)** | 0.001 |
| LPA | SB | 40 | -0.82 (-1.27, -0.37)** | -0.14 (-0.22, -0.06)** | 0.001 |
| LPA | SB | 45 | -0.92 (-1.42, -0.41)** | -0.16 (-0.25, -0.07)** | 0.001 |
| LPA | SB | 50 | -1.01 (-1.56, -0.45)** | -0.18 (-0.27, -0.08)** | 0.001 |
| LPA | SB | 55 | -1.10 (-1.71, -0.50)** | -0.19 (-0.30, -0.09)** | 0.001 |
| LPA | SB | 60 | -1.19 (-1.84, -0.54)** | -0.21 (-0.32, -0.09)** | < .001 |
| LPA | SLP | 5 | -0.08 (-0.16, -0.00)* | -0.01 (-0.03, -0.00)* | 0.047 |
| LPA | SLP | 10 | -0.16 (-0.32, -0.00)* | -0.03 (-0.06, -0.00)* | 0.048 |
| LPA | SLP | 15 | -0.24 (-0.48, -0.00)* | -0.04 (-0.08, -0.00)* | 0.049 |
| LPA | SLP | 20 | -0.32 (-0.63, -0.00)* | -0.06 (-0.11, -0.00)* | 0.05 |
| LPA | SLP | 25 | -0.39 (-0.78, -0.00)* | -0.07 (-0.14, -0.00)* | 0.051 |
| LPA | SLP | 30 | -0.47 (-0.93, -0.00)* | -0.08 (-0.16, -0.00)* | 0.052 |
| LPA | SLP | 35 | -0.54 (-1.07, 0.00) | -0.09 (-0.19, 0.00) | 0.054 |
| LPA | SLP | 40 | -0.61 (-1.21, 0.00) | -0.11 (-0.21, 0.00) | 0.055 |
| LPA | SLP | 45 | -0.67 (-1.35, 0.01) | -0.12 (-0.23, 0.00) | 0.056 |
| LPA | SLP | 50 | -0.74 (-1.49, 0.01) | -0.13 (-0.26, 0.00) | 0.057 |
| LPA | SLP | 55 | -0.80 (-1.62, 0.02) | -0.14 (-0.28, 0.00) | 0.059 |
| LPA | SLP | 60 | -0.87 (-1.76, 0.02) | -0.15 (-0.30, 0.00) | 0.06 |
| MVPA | LPA | 5 | -0.23 (-0.44, -0.03)* | -0.04 (-0.08, -0.01)* | 0.031 |
| MVPA | LPA | 10 | -0.43 (-0.82, -0.04)* | -0.07 (-0.14, -0.01)* | 0.039 |
| MVPA | LPA | 15 | -0.58 (-1.14, -0.03)* | -0.10 (-0.20, -0.00)* | 0.049 |
| MVPA | LPA | 20 | -0.72 (-1.43, 0.00) | -0.12 (-0.25, 0.00) | 0.06 |
| MVPA | LPA | 25 | -0.82 (-1.69, 0.05) | -0.14 (-0.29, 0.01) | 0.074 |
| MVPA | LPA | 30 | -0.91 (-1.92, 0.10) | -0.16 (-0.33, 0.02) | 0.09 |
| MVPA | LPA | 35 | -0.98 (-2.13, 0.17) | -0.17 (-0.37, 0.03) | 0.109 |
| MVPA | LPA | 40 | -1.03 (-2.32, 0.26) | -0.18 (-0.40, 0.04) | 0.13 |
| MVPA | LPA | 45 | -1.07 (-2.49, 0.35) | -0.19 (-0.43, 0.06) | 0.155 |
| MVPA | LPA | 50 | -1.09 (-2.64, 0.46) | -0.19 (-0.46, 0.08) | 0.183 |
| MVPA | LPA | 55 | -1.10 (-2.79, 0.58) | -0.19 (-0.48, 0.10) | 0.216 |
| MVPA | LPA | 60 | -1.10 (-2.91, 0.72) | -0.19 (-0.51, 0.12) | 0.253 |
| MVPA | SB | 5 | -0.35 (-0.52, -0.17)** | -0.06 (-0.09, -0.03)** | < .001 |
| MVPA | SB | 10 | -0.65 (-0.98, -0.32)** | -0.11 (-0.17, -0.06)** | < .001 |
| MVPA | SB | 15 | -0.93 (-1.40, -0.46)** | -0.16 (-0.24, -0.08)** | < .001 |
| MVPA | SB | 20 | -1.18 (-1.77, -0.59)** | -0.20 (-0.31, -0.10)** | < .001 |
| MVPA | SB | 25 | -1.41 (-2.12, -0.71)** | -0.25 (-0.37, -0.12)** | < .001 |
| MVPA | SB | 30 | -1.63 (-2.44, -0.82)** | -0.28 (-0.42, -0.14)** | < .001 |
| MVPA | SB | 35 | -1.83 (-2.73, -0.93)** | -0.32 (-0.47, -0.16)** | < .001 |
| MVPA | SB | 40 | -2.02 (-3.01, -1.02)** | -0.35 (-0.52, -0.18)** | < .001 |
| MVPA | SB | 45 | -2.20 (-3.28, -1.12)** | -0.38 (-0.57, -0.19)** | < .001 |
| MVPA | SB | 50 | -2.37 (-3.52, -1.21)** | -0.41 (-0.61, -0.21)** | < .001 |
| MVPA | SB | 55 | -2.53 (-3.76, -1.30)** | -0.44 (-0.65, -0.23)** | < .001 |
| MVPA | SB | 60 | -2.69 (-3.99, -1.38)** | -0.47 (-0.69, -0.24)** | < .001 |
| MVPA | SLP | 5 | -0.32 (-0.50, -0.14)** | -0.06 (-0.09, -0.02)** | 0.001 |
| MVPA | SLP | 10 | -0.60 (-0.94, -0.26)** | -0.10 (-0.16, -0.05)** | 0.001 |
| MVPA | SLP | 15 | -0.85 (-1.33, -0.37)** | -0.15 (-0.23, -0.06)** | 0.001 |
| MVPA | SLP | 20 | -1.08 (-1.68, -0.47)** | -0.19 (-0.29, -0.08)** | 0.001 |
| MVPA | SLP | 25 | -1.28 (-2.00, -0.56)** | -0.22 (-0.35, -0.10)** | 0.001 |
| MVPA | SLP | 30 | -1.47 (-2.30, -0.64)** | -0.25 (-0.40, -0.11)** | 0.001 |
| MVPA | SLP | 35 | -1.64 (-2.57, -0.71)** | -0.28 (-0.45, -0.12)** | 0.001 |
| MVPA | SLP | 40 | -1.80 (-2.83, -0.78)** | -0.31 (-0.49, -0.14)** | 0.001 |
| MVPA | SLP | 45 | -1.96 (-3.07, -0.84)** | -0.34 (-0.53, -0.15)** | 0.001 |
| MVPA | SLP | 50 | -2.10 (-3.30, -0.90)** | -0.36 (-0.57, -0.16)** | 0.001 |
| MVPA | SLP | 55 | -2.23 (-3.51, -0.95)** | -0.39 (-0.61, -0.17)** | 0.001 |
| MVPA | SLP | 60 | -2.36 (-3.72, -1.00)** | -0.41 (-0.64, -0.17)** | 0.002 |
| SB | LPA | 5 | 0.11 (0.05, 0.18)** | 0.02 (0.01, 0.03)** | 0.001 |
| SB | LPA | 10 | 0.23 (0.10, 0.36)** | 0.04 (0.02, 0.06)** | 0.001 |
| SB | LPA | 15 | 0.34 (0.14, 0.54)** | 0.06 (0.03, 0.09)** | 0.001 |
| SB | LPA | 20 | 0.46 (0.19, 0.73)** | 0.08 (0.03, 0.13)** | 0.001 |
| SB | LPA | 25 | 0.59 (0.24, 0.93)** | 0.10 (0.04, 0.16)** | 0.001 |
| SB | LPA | 30 | 0.71 (0.29, 1.13)** | 0.12 (0.05, 0.20)** | 0.001 |
| SB | LPA | 35 | 0.84 (0.35, 1.34)** | 0.15 (0.06, 0.23)** | 0.001 |
| SB | LPA | 40 | 0.98 (0.40, 1.55)** | 0.17 (0.07, 0.27)** | 0.001 |
| SB | LPA | 45 | 1.11 (0.45, 1.78)** | 0.19 (0.08, 0.31)** | 0.001 |
| SB | LPA | 50 | 1.26 (0.51, 2.01)** | 0.22 (0.09, 0.35)** | 0.001 |
| SB | LPA | 55 | 1.41 (0.56, 2.25)** | 0.24 (0.10, 0.39)** | 0.001 |
| SB | LPA | 60 | 1.56 (0.62, 2.50)** | 0.27 (0.11, 0.43)** | 0.001 |
| SB | MVPA | 5 | 0.40 (0.19, 0.60)** | 0.07 (0.03, 0.10)** | < .001 |
| SB | MVPA | 10 | 0.87 (0.42, 1.32)** | 0.15 (0.07, 0.23)** | < .001 |
| SB | MVPA | 15 | 1.46 (0.70, 2.21)** | 0.25 (0.12, 0.38)** | < .001 |
| SB | MVPA | 20 | 2.23 (1.06, 3.40)** | 0.39 (0.18, 0.59)** | < .001 |
| SB | MVPA | 25 | 3.39 (1.60, 5.17)** | 0.59 (0.28, 0.90)** | 0.001 |
| SB | MVPA | 30 | 5.83 (2.72, 8.95)** | 1.01 (0.47, 1.55)** | 0.001 |
| SB | SLP | 5 | 0.03 (-0.02, 0.07) | 0.00 (-0.00, 0.01) | 0.222 |
| SB | SLP | 10 | 0.05 (-0.03, 0.14) | 0.01 (-0.01, 0.02) | 0.225 |
| SB | SLP | 15 | 0.08 (-0.05, 0.20) | 0.01 (-0.01, 0.04) | 0.228 |
| SB | SLP | 20 | 0.10 (-0.07, 0.27) | 0.02 (-0.01, 0.05) | 0.231 |
| SB | SLP | 25 | 0.13 (-0.08, 0.34) | 0.02 (-0.01, 0.06) | 0.234 |
| SB | SLP | 30 | 0.15 (-0.10, 0.41) | 0.03 (-0.02, 0.07) | 0.238 |
| SB | SLP | 35 | 0.18 (-0.12, 0.48) | 0.03 (-0.02, 0.08) | 0.241 |
| SB | SLP | 40 | 0.20 (-0.14, 0.54) | 0.04 (-0.02, 0.09) | 0.244 |
| SB | SLP | 45 | 0.23 (-0.16, 0.61) | 0.04 (-0.03, 0.11) | 0.247 |
| SB | SLP | 50 | 0.25 (-0.18, 0.68) | 0.04 (-0.03, 0.12) | 0.251 |
| SB | SLP | 55 | 0.27 (-0.20, 0.75) | 0.05 (-0.03, 0.13) | 0.254 |
| SB | SLP | 60 | 0.30 (-0.22, 0.82) | 0.05 (-0.04, 0.14) | 0.258 |
| SLP | LPA | 5 | 0.09 (0.00, 0.17)* | 0.01 (0.00, 0.03)* | 0.045 |
| SLP | LPA | 10 | 0.17 (0.01, 0.34)* | 0.03 (0.00, 0.06)* | 0.044 |
| SLP | LPA | 15 | 0.26 (0.01, 0.52)* | 0.05 (0.00, 0.09)* | 0.043 |
| SLP | LPA | 20 | 0.36 (0.02, 0.70)* | 0.06 (0.00, 0.12)* | 0.042 |
| SLP | LPA | 25 | 0.46 (0.03, 0.89)* | 0.08 (0.00, 0.15)* | 0.041 |
| SLP | LPA | 30 | 0.56 (0.03, 1.08)* | 0.10 (0.01, 0.19)* | 0.04 |
| SLP | LPA | 35 | 0.66 (0.04, 1.28)* | 0.11 (0.01, 0.22)* | 0.039 |
| SLP | LPA | 40 | 0.77 (0.05, 1.49)* | 0.13 (0.01, 0.26)* | 0.038 |
| SLP | LPA | 45 | 0.88 (0.07, 1.70)* | 0.15 (0.01, 0.30)* | 0.037 |
| SLP | LPA | 50 | 1.00 (0.08, 1.93)* | 0.17 (0.01, 0.33)* | 0.036 |
| SLP | LPA | 55 | 1.13 (0.09, 2.16)* | 0.20 (0.02, 0.37)* | 0.035 |
| SLP | LPA | 60 | 1.26 (0.11, 2.40)* | 0.22 (0.02, 0.42)* | 0.034 |
| SLP | MVPA | 5 | 0.37 (0.17, 0.58)** | 0.06 (0.03, 0.10)** | 0.001 |
| SLP | MVPA | 10 | 0.82 (0.36, 1.28)** | 0.14 (0.06, 0.22)** | 0.001 |
| SLP | MVPA | 15 | 1.38 (0.62, 2.14)** | 0.24 (0.11, 0.37)** | 0.001 |
| SLP | MVPA | 20 | 2.13 (0.95, 3.30)** | 0.37 (0.17, 0.57)** | 0.001 |
| SLP | MVPA | 25 | 3.26 (1.46, 5.05)** | 0.57 (0.25, 0.88)** | 0.001 |
| SLP | MVPA | 30 | 5.68 (2.56, 8.80)** | 0.99 (0.44, 1.53)** | 0.001 |
| SLP | SB | 5 | -0.03 (-0.07, 0.02) | -0.00 (-0.01, 0.00) | 0.216 |
| SLP | SB | 10 | -0.05 (-0.14, 0.03) | -0.01 (-0.02, 0.01) | 0.213 |
| SLP | SB | 15 | -0.08 (-0.20, 0.05) | -0.01 (-0.04, 0.01) | 0.21 |
| SLP | SB | 20 | -0.11 (-0.27, 0.06) | -0.02 (-0.05, 0.01) | 0.207 |
| SLP | SB | 25 | -0.13 (-0.34, 0.07) | -0.02 (-0.06, 0.01) | 0.204 |
| SLP | SB | 30 | -0.16 (-0.41, 0.09) | -0.03 (-0.07, 0.02) | 0.201 |
| SLP | SB | 35 | -0.19 (-0.48, 0.10) | -0.03 (-0.08, 0.02) | 0.198 |
| SLP | SB | 40 | -0.22 (-0.55, 0.11) | -0.04 (-0.09, 0.02) | 0.195 |
| SLP | SB | 45 | -0.25 (-0.62, 0.13) | -0.04 (-0.11, 0.02) | 0.193 |
| SLP | SB | 50 | -0.27 (-0.69, 0.14) | -0.05 (-0.12, 0.02) | 0.19 |
| SLP | SB | 55 | -0.30 (-0.75, 0.15) | -0.05 (-0.13, 0.03) | 0.187 |
| SLP | SB | 60 | -0.33 (-0.82, 0.16) | -0.06 (-0.14, 0.03) | 0.184 |
| ^2^ The model was adjusted for sex, age, BMI, education level, socioeconomic status ,number of chronic diseases; sd: Standard Deviation; * *p*<0.05, ** *p*<0.01 | | | | | |
